# The Influence of Health Education on Vaccination Coverage and Knowledge of the School Population Related to Vaccination and Infection Caused by the Human Papillomavirus

**DOI:** 10.3390/vaccines12111222

**Published:** 2024-10-28

**Authors:** Ivana Kotromanović Šimić, Vesna Bilić-Kirin, Maja Miskulin, Darko Kotromanović, Marija Olujić, Jelena Kovacevic, Danijela Nujić, Nika Pavlovic, Ivan Vukoja, Ivan Miskulin

**Affiliations:** 1 Faculty of Medicine Osijek, Josip Juraj Strossmayer University of Osijek, 31000 Osijek, Croatia; ivsimic@mefos.hr (I.K.Š.); vesna.bilic.kirin@gmail.com (V.B.-K.); maja.miskulin@mefos.hr (M.M.); kotromanovic93@gmail.com (D.K.); molujic@mefos.hr (M.O.); dr.kovacevic.jelena@gmail.com (J.K.); danijela.nujic@mefos.hr (D.N.); nika.felicita@gmail.com (N.P.); iv.vukoja@gmail.com (I.V.); 2 Institute of Public Health for the Osijek-Baranja County, 31000 Osijek, Croatia; 3 Oncology Clinic, UHC Osijek, 31000 Osijek, Croatia; 4 Ophthalmology Polyclinic Dr. Balog, 31000 Osijek, Croatia; 5 Institute of Emergency Medicine of the Vukovar-Srijem County, 32100 Vinkovci, Croatia; 6 County General Hospital Požega, 34000 Požega, Croatia

**Keywords:** health education, human papillomavirus (HPV), knowledge, vaccination

## Abstract

Background/Objectives: Human papillomavirus (HPV) is a causative agent of infections and cancers of the reproductive and digestive tract, and vaccination is the most effective prevention method. This research aimed to assess the impact of health education on vaccination coverage and knowledge of the school population related to HPV infection and vaccination. Methods: This non-randomized clinical trial included 170 participants out of the 221 students in the generation of 2022/2023, who were divided into a group of Subjects and Controls and who self-assessed their knowledge and tested knowledge at four and two time points, respectively. Results: The study examined whether there is a difference in the share of vaccinated students of the entire generation compared to the previous generation (62% vs. 47%). The self-assessment and assessment of one’s knowledge in the group of subjects was significantly lower before education than during other time points during the research. At “moment zero”, there was no significant difference in the knowledge assessment between the groups. However, after 8 weeks, the knowledge assessment was significantly higher in the Subject group. Conclusions: The results suggest a positive impact of health education, which may contribute to raising awareness about the importance of prevention and vaccination against HPV.

## 1. Introduction

Human papillomavirus (HPV) is one of the most prevalent sexually transmitted infections (STIs), so widespread and easily contracted that nearly all sexually active individuals will encounter the virus at some point in their lives [1]. HPV is primarily transmitted through unprotected sexual activity, including vaginal, anal, and oral sex, but it can also spread through skin-to-skin contact during non-penetrative sexual behaviors, such as hand-to-genital contact. While sexual contact is the most common route of transmission, HPV can also be passed from an infected mother to her child during childbirth, though this occurs less frequently [1,2,3,4,5]. Moreover, HPV is also diagnosed in children and adolescents who have not been sexually active, leading researchers to investigate additional transmission methods [1]. 

There are over 200 genotypes of human papillomavirus (HPV), with approximately 40 of these affecting the anogenital tract. These genotypes can be categorized into two main groups: low oncogenic risk and high oncogenic risk. High-risk HPV serotypes are associated with the development of various cancers. These serotypes can induce changes in the epithelium, leading to lesions on the skin and mucous membranes, particularly in the anogenital area. They are linked to several types of cancer, including anal cancer, vulvar cancer, vaginal cancer, penile cancer, oropharyngeal cancer, oral cavity cancer, and laryngeal cancer. On the other hand, low-risk HPV serotypes, such as types 6 and 11, play a significant role as well. While they are not typically associated with cancer, they are responsible for approximately 90% of genital warts [6].

Luckily, most of the HPV infections are asymptomatic and pass spontaneously [3,5]. A decrease in HPV serotype 6, 11, 16, and 18 infection, genital warts, low-grade cytological cervical abnormalities, and histologically proven cervical abnormalities has been observed after an HPV vaccine introduction [7]. There is no specific “cure” for the HPV infection, which is why it is necessary to prevent the infection, and this action can be achieved by vaccination against HPV. Results of randomized trials confirmed a high safety profile of HPV vaccines [8]. Vaccination against HPV with today’s nine-valent vaccine is intended to protect against precancerous lesions, cancers, and genital warts associated with HPV infection [3,4,5,7,8,9]. Research has shown that the largest number of new HPV infections occur in people between the ages of 14 and 25 who can already become infected during the first sexual intercourse with an infected partner [2,3,5,7,8].

Vaccinating both girls and boys before potential exposure to specific types of HPV linked to cancer is crucial. Research indicates that the greatest benefits of vaccination are observed in girls who receive the vaccine prior to HPV exposure, particularly in countries like Australia that have achieved high vaccination coverage rates [3,5,7]. Since 2006, many European countries have incorporated HPV vaccination against the most common low- and high-risk subtypes into their publicly funded national immunization programs [6,9]. Currently, the majority of these countries utilize nine-valent vaccines, which have been in use in Europe and Croatia since 2015 and in the United States since 2014. The Food and Drug Administration, European Medicines Agency, and Agency of Medicinal Products and Medical Devices of Croatia have approved this vaccine for use in boys and girls aged 9 and older [2,3,5,10,11,12,13,14].

In 2007, HPV vaccination was first introduced in Croatia with the bi-valent vaccine as optional and recommended. From the school year 2016/2017, vaccination became available free of charge for girls and boys in the eighth grade of primary school. Since 2015, the only HPV vaccine used for vaccination in Croatia is the 9-valent HPV vaccine instead of the quadrivalent or bivalent vaccine used until then [3,5,15]. With the shift of vaccination primarily to the 8th grade of primary school, school doctors have become the only vaccinators against HPV infection. Vaccination against HPV infection is the only voluntary and free vaccine in the Republic of Croatia [3,5]. Croatia is one of the countries in Europe that started vaccinating children at the latest, given that the average age of students in the 8th grade is from 14 to 15 years [16]. Therefore, to adjust with other countries, from 1 January 2023, vaccination against diseases caused by HPV infection became available at the national level to girls and boys from the 5th grade of primary school based on the recommendation of the Croatian Institute of Public Health and the Croatian Society of School and University Medicine through the Program of Immunization, Seroprophylaxis, and Chemoprophylaxis for special population groups and individuals at increased risk of certain infections. The aforementioned regulation enables vaccination at individual request at a younger age, i.e., for all persons from 9 to 25 years of age [17].

The school year 2023/2024 was the first complete school year during which all the students of the fifth, sixth, seventh, and eighth grades of primary school were organized to be invited to vaccinate against HPV infection as a part of the prescribed and estimated preventive activities, examinations, and measures with the school doctor in charge [16]. It is extremely important to vaccinate boys as well as girls because, despite the frequent prejudice that boys are only carriers of the virus that do not suffer the consequences of infection, it has been proven that they can also suffer from HPV-related diseases. All students are vaccinated according to the following scheme: girls and boys up to and including 14 years of age receive two doses of the vaccine 6 months apart (0, 6); while students of 15 or more years of age receive the vaccine in three doses, with a recommended interval of two months between the first and second dose, and four months between the second and third dose (0, 2, 6). The intervals between doses can be longer, but it is recommended to receive all three doses of the vaccine within 12 months [8,9,14,17]. Research has shown that most of the new HPV infections occur in people between the ages of 14 and 25 who can become infected during their first sexual intercourse with an infected partner [5,8].

There is still a shortage of sources about the coverage of HPV vaccination and the reasons for the assumed, relatively low vaccination rate against HPV in Croatia, as well as the level of knowledge and the influence of health education on the knowledge of the school population about vaccination and infection caused by HPV.

The uniqueness of our study is manifested, above all, in the fact that, until now, no research has been conducted on the influence of health education on vaccination coverage and knowledge of the school population related to the vaccination and infection caused by the Human Papillomavirus in the Republic of Croatia. Furthermore, until now, there is no research in which data on the vaccination coverage against HPV among school children in the Republic of Croatia, or Osijek-Baranja county, has been published.

Therefore, the objectives of our research were to examine whether there is an impact of health education on students’ knowledge about vaccination and infection caused by HPV, as well as whether there are differences in students’ knowledge about vaccination and infection caused by HPV, depending on the participation in health education about HPV infection and vaccination. Finally, we investigated and compared the proportion of vaccinated eighth-grade students against HPV from two generations of the selected elementary schools in Osijek-Baranja County, and we investigated whether there is a difference in the proportion of vaccinated eighth-grade students against HPV depending on the participation in health education about HPV infection and vaccination.

## 2. Materials and Methods

This research was organized as a non-randomized clinical trial that was conducted throughout the school year 2022/2023. Participants in the research were students in the final grade of four elementary schools in Osijek-Baranja County. Since the participating students were under the age of 18, their parents/guardians signed a consent form for their participation in this research. The conduct of this research was approved by the ethics committee of the Faculty of Medicine Osijek (date of approval: 10 September 2021, Class: 602-04/21-08/7, Reg. No.: 2158-61-07-21-170) and the Teaching Institute of Public Health of Osijek-Baranja County (date of approval: 8 February 2021, Class: 035-01/21-01/14, Reg. No.: 391-21-4).

Inclusive criteria for participation in the research were age (between 14 and 15 years old), attendance in the 8th grade of primary school, and parental consent to participate in the research. The exclusive criterion was the disagreement of parents/guardians with the participation of students in the research. At the beginning of the research, the participants were divided into two groups (Subjects and Controls), and the only criterion for the division was an equal number of participants in each group.

Before starting this research, using G*Power (ver. 3.1.9.4), we calculated the minimum required sample size to observe a mean effect in the difference in numerical variables between two independent groups of subjects, with a significance level of 0.05 and a power of 0.9, which was 156 subjects, i.e., a minimum of 78 subjects per group [18]. Therefore, our research included 170 students 79 (46.5%) in the Subject group and 91 (53.5%) in the Control group.

We examined the knowledge about vaccination and infection caused by HPV with the help of a survey questionnaire created for this research. The aforementioned survey questionnaire was completely anonymous since each student participant independently designed the code that they used when completing the survey questionnaire. Subjects completed the questionnaire at four different time points during the research: before the health education was completed (“moment zero”), immediately after the health education was completed, four weeks, and eight weeks after the health education was completed. The Controls completed the survey questionnaire at only two time points: at “moment zero” and eight weeks later. Health education on vaccination and infection caused by HPV was conducted through a short lecture given by a school doctor. The first part of the questionnaire contained questions related to the sociodemographic characteristics of the participating students and sources of information about HPV vaccination and infection while the second part of the questionnaire contained claims related to the self-assessment of their knowledge and the assessment of knowledge about vaccination and infection caused by HPV. When completing the second part of the questionnaire, participants were first asked to answer three questions related to the self-assessment of their knowledge about HPV and cervical cancer, the severity of cervical cancer, and the amount of knowledge about the HPV vaccine. The knowledge about HPV and the vaccine against HPV was originally taken from a research study conducted by Caskey et al. and translated into Croatian [19,20]. The Croatian version of this questionnaire, which was used in this research, was taken from research conducted by Delač and Korajlija, with their written permission [20]. The questionnaire contained three questions on the self-assessment of HPV and vaccine knowledge, eight statements on HPV knowledge and infection, and six questions on HPV vaccine knowledge. The total score was calculated as the sum of all correct answers where each correct answer scored one point. The possible score range was from 0 to 14, and a higher score indicated greater knowledge of HPV infection, vaccine, and HPV vaccination [19,20].

Data on the percentage of HPV vaccination of the entire generation of students in the observed school year 2022/2023 were obtained from the medical records of the students from the corresponding school doctor. The obtained data were compared with the data on HPV vaccination of the previous generation of all students of the same primary schools. Incomplete questionnaires were not included in our analysis. Almost one complete class of students was missing on the day when our measurements occurred due to the World Football Cup. Thus, the Minister of Education of the Republic of Croatia allowed school children to miss classes on the day when the Croatian national football team played a crucial match at the World Football Championship. We did not expect some specific factors that impacted these students to not complete a questionnaire, and we did not expect bias in the results.

Using absolute and relative frequencies, categorical data were presented. Differences in categorical variables were tested by Chi-square (2) and Fisher’s exact test. Differences in dependent categorical variables were tested with the McNemar–Bowker test. The normality of the distribution of continuous variables was tested with the Shapiro–Wilk test. Continuous data are described by measures of mean and dispersion depending on the normality of the distribution. Differences in continuous variables were tested with the Mann–Whitney U test (Hodges–Lehmann median difference and a 95% CI), the Kruskal–Wallis test, and the Friedman test between measurements (Post hoc Test Conover). The Bonferroni correction was used for multiple comparisons. The Bonferroni correction was used for multiple comparisons. All *p* values are two-sided. The significance level was set at alpha (α) = 0.05. For statistical analysis, we used the statistical programs MedCalc^®^ Statistical Software version 22.018 (MedCalc Software Ltd., Ostend, Belgium; https://www.medcalc.org; accessed on 6 September 2024) and SPSS 23.0 (Published 2015 IBM Armonk, IBM Corp., New York, NY, USA) [21,22].

## 3. Results

### 3.1. Basic Characteristics of the Participating Students

This research included 170 students; 79 (46.5%) of them formed a Subject group and 91 (53.5%) formed a Control group. The mean age of student participants was 14 years (SD—standard deviations 0.5). The Control group (without health education completed) consisted of 48 (28.2%) students from Retfala Elementary School and 43 (25.3%) students from I. Filipović Elementary School. The Subject group (with health education completed) consisted of 41 (24.1%) students from Višnjevac Elementary School and 38 (22.4%) students from Josipovac Elementary School. There was no significant difference in age and sex of the subjects between the groups (Table 1 and Table 2).

A total of 109 (64.1%) student participants were vaccinated, with no significant difference between groups (Table 3). However, significantly more female than male students participating in this study were vaccinated (χ^2^ tests, *p* = 0.02) (Table 4).

### 3.2. Student Vaccination Rate in the School Year 2021/2022 and 2022/2023

In the school year 2021/2022, out of a total of 196 students, 92 (47%) students were vaccinated. In the school year 2022/2023, out of a total of 221 students, 137 (62%) students were vaccinated, which is an increase in the share of vaccinated students by 15% (Table 5).

In this research, out of a total of 170 student participants, 109 (64%) were vaccinated. The proportion of vaccinated students in the Subject group and the Control group is approximately equal (Table 6).

**Figure 1:**
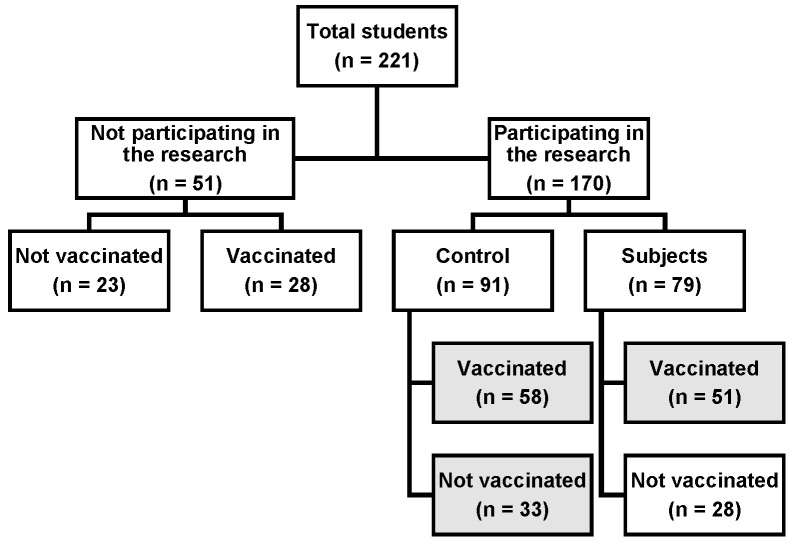
Distribution of students by participation in research and HPV vaccination.

Figure 1 shows the distribution of students according to participation in research and vaccination against HPV.

### 3.3. Self-Assessment of HPV Infection Knowledge and HPV Vaccination

All participants assessed their knowledge of HPV infection and HPV vaccination through three questions. The Control group at “moment zero” and 8 weeks later and the Subject group at “moment zero” (i.e., before health education), immediately after health education, 4 weeks later and 8 weeks later. At “moment zero”, in both the Control group and Subject group, most students stated that their knowledge of HPV and cervical cancer, the severity of cervical cancer, or the HPV vaccine, was insufficient or sufficient (50% to 80% of the students). The self-assessment of one’s knowledge at “moment zero” was significantly higher in the Control group compared to the Subjects (Mann–Whitney U test, *p* = 0.04) (Table 7).

The self-assessment of one’s knowledge in the Subject group was significantly lower before the education compared to the time immediately after the education or 4 and 8 weeks later. The self-assessment of one’s knowledge was significantly lowest before education while the self-assessment of knowledge immediately after education was significantly highest (Friedman test, *p* < 0.001). The significantly higher self-assessment of knowledge was immediately after the education (median grade 11 vs. 9 and 11 vs. 10, respectively) compared to 4 and 8 weeks after the education (Friedman test, *p* < 0.001) (Table 8).

Eighth weeks after the education, it was noted that a smaller number of the students in the Control group, compared to the Subjects, stated that their knowledge of HPV and cervical cancer, the severity of cervical cancer, and the HPV vaccine was very good or excellent (Table 9).

The self-assessment of one’s knowledge after 8 weeks was significantly better among the students who have completed education (Subjects) compared to the Control group of students (median 10 vs. 6) (Mann–Whitney U test, *p* < 0.001) (Table 10).

### 3.4. Assessment of HPV Infection Knowledge and HPV Vaccination

The knowledge of HPV infection and vaccination was assessed by a 14-question scale taken from a research study conducted by Caskey et al. During the testing at “moment zero”, most students, 145 (85.3%), gave the correct answer that a girl/woman can become infected with HPV during intercourse, 134 (78.8%) students answered correctly that HPV infection can cause cervical cancer, or that girls/women vaccinated against HPV must use condoms during intercourse. The correct answer to the claim that HPV infection can be diagnosed during a gynecological examination or pelvic examination was given by 131 (77.5%) students, and 127 (74.7%) answered correctly that women vaccinated against HPV should have a PAP smear. There was no significant difference in knowledge between the Subject and Control group at “moment zero” (Table 11).

Correct answers of the Subjects related to knowledge, at three assessment points, as well as the significance in the distribution of the Subjects by correct answers given, are shown in Table 12.

The Subject’s knowledge was significantly lower before the education, compared to all other assessment points (Friedman test, *p* < 0.001) (Table 13).

At the last point of assessment, i.e., 8 weeks after the education, the knowledge of the Subjects was significantly better compared to the knowledge of the students from the Control group, given the question that, if the findings of the PAP smear were normal, the girl/woman was not infected with HPV (χ^2^ test, *p* < 0.001); when asked whether HPV infection can cause genital warts (χ^2^ test, *p* = 0.002); whether HPV infection can be diagnosed by a blood test (χ^2^ test, *p* = 0.005); whether the HPV vaccine protects against cervical cancer (χ^2^ test, *p* = 0.03); and when asked whether girls/women vaccinated against HPV can worry less about whether they will be infected with sexually transmitted diseases (χ^2^ test, *p* = 0.009). Also, in the eighth week after the education, the knowledge of students who underwent education (Subjects) was significantly better assessed compared to the Control group (median 11 vs. 9) (Mann–Whitney U test, *p* < 0.001) (Table 14).

## 4. Discussion

Despite the availability of an HPV vaccine for over a decade, HPV continues to be a significant cause of morbidity and mortality worldwide, particularly in relation to cervical cancer, which is closely linked to HPV infection. This cancer is preventable through vaccination and can be effectively treated if detected early. Cervical cancer ranks as the fourth most common cancer among women globally, underscoring the critical need for robust vaccination programs to achieve high coverage rates [6,7,13,23]. In response to the rising incidence of cervical cancer, the World Health Organization (WHO) launched a global initiative in November 2020 aimed at eliminating cervical cancer as a public health issue in the 21st century. The initiative’s goals include vaccinating at least 90% of girls, screening 70% of women using high-performance tests, and treating at least 90% of identified precancerous lesions and invasive cancers [24]. In Croatia, there are approximately 1.80 million women aged 15 and older at risk of cervical cancer. Current estimates suggest that 336 women are diagnosed with cervical cancer each year, resulting in about 150 deaths. Cervical cancer is the tenth most common cancer among women in Croatia and ranks third among women aged 15 to 44. It is estimated that around 18% of women in the general population are currently infected with HPV types 16 or 18, which are responsible for approximately 82.9% of invasive cervical cancers [3,25,26]. As there is no specific cure for HPV infection, prevention through vaccination is essential. Currently, 125 countries have included the HPV vaccine in their national immunization programs for girls, while 47 countries offer it for both girls and boys. According to WHO estimates from 2022, only about 15% of girls worldwide are fully vaccinated against HPV, with regional coverage varying significantly: Africa (22%), America (52%), Eastern Mediterranean (0%), Europe (32%), Southeast Asia (3%) and Western Pacific (3%). Data on vaccination coverage in Croatia are still unavailable [26,27,28].

The exact coverage of HPV vaccination varies by country in Europe and by sex. The vaccination coverage ranges from 64% in Iceland to 9% in Bulgaria, while the European average vaccination rate was 64%. Countries with a vaccination rate above 90% were Iceland, Portugal and Norway, while Bulgaria was the only country with a vaccination rate below 10%. In almost all countries, girls had a higher HPV vaccination rate than boys [16].

The potential reason for different vaccine coverage may be due to differences in national vaccination programs across European countries. In Austria, Belgium, Croatia, Cyprus, Czechia, Denmark, Finland, France, Germany, Greece, Hungary, Iceland, Ireland, Italy, Latvia, Lithuania, Luxembourg, Malta, Netherlands, Norway, Poland, Portugal, Romania, Slovak Republic, Slovenia, Spain, and Sweden, all children were included in the vaccination strategy, but, in Bulgaria and Estonia, only girls were included in the vaccination strategy. The target age for primary vaccination varies by country, from 9 years old in Austria, Germany, Greece, Luxembourg, and Malta to 18 years old in Romania or 15 years old in Croatia and Slovak Republic. The catch-up period also varies by country from 12 to 18 years old in the Wallonia and Brussels region, up to 25 years old in Ireland, or 26 years old in the Netherlands. Some of the countries do not have catch-up at all, such as Bulgaria, Czechia, Estonia, Hungary, Iceland, Latvia, Lithuania, Malta, Romania, Slovak Republic, Slovenia, and Spain. The majority of the countries had vaccine registries, with the exception of Austria, Bulgaria, Croatia, Cyprus, Czechia, France, Germany, Greece, Poland and the Slovak Republic. Countries with national school-based vaccination programs were Wallonia (Brussels), Croatia, Cyprus, Estonia, Finland, France, Hungary, Iceland, Ireland, Norway, Slovenia, Spain, and Sweden. The catch-up period also varies by country, from 12 to 18 years old in the Wallonia and Brussels region, up to 25 years old in Ireland, or 26 years old in the Netherlands [16].

HPV vaccination coverage rates (HPV-VCRs) in Croatia are not regularly monitored, unlike mandatory vaccinations that are included in the national immunization programs during childhood and adolescence, where VCRs are set at 95% for all vaccinated cohorts. There is still much-needed information about the coverage of vaccination against HPV missing because the data are still publicly unavailable [10]. Unlike other European countries, there is no vaccination registry in Croatia for HPV, and, therefore, HPV-VCRs in Croatia are still unknown, but they are considered low compared to other European countries and lagging behind the goal and recommendations set by WHO [16,26,27,28]. Although there are still no accurate data on HPV-VCR at the national level, there are data on HPV-VCR at the level of Osijek-Baranja County for both sexes of elementary school students who predominantly completed the eighth grade in the observed school years. HPV-VCR ranged from 40% in the school year 2018/2019 and the school year 2019/2020, 42% in the school year 2020/2021, 38% in the school year 2021/2022, 56% in the school year 2022/2023, and to 65% in the 2023/2024 school year [29].

The total vaccination coverage of eighth-grade students in Osijek-Baranja County in the school year 2022/2023 was 56%, which is an increase of 18% compared to the previous school year. Although the data on the proportion of vaccinated eighth-grade students (65%) are currently incomplete, due to ongoing systematic examination, an increase in the proportion of vaccinated primary school students is visible again, which indicates the fact that this school year will end with the highest proportion of vaccinated primary school students so far in the Osijek-Baranja County. The collected results correspond to the results obtained from the Krnić et al. study [15] and show a positive upward trend in the coverage of HPV vaccination among the target population. However, these results are still below the recommended levels, which is why it is necessary to continue working on increasing vaccination coverage to get as close as possible to the WHO target [7,23,24,27]. According to the data of the Croatian Institute for Public Health (CIPH) on HPV immunization of the school population from the school year 2018/2019 to 2021/2022, considering the number of applied HPV vaccine doses, a positive trend in increasing the number of HPV vaccine doses consumed among primary school students is observed, both at the state level (17,582 vs. 20,766 vs. 26,782 vs. 24,574) and at the level of the Osijek-Baranja County (1419 vs. 1605. vs. 1786. vs. 1712). The female–male ratio among the vaccinated was in the range from 2.3 in 2018 to 1.7 in 2021 per the CIPH Communicable Diseases Epidemiology Division data. The vaccination rate against HPV with at least one dose of the vaccine, among children born in 2008 who predominantly completed the eighth grade in the school year 2022/2023 (date up to 31st August 2023) at the state level, was 51.1% (9964) girls and 34.3% (6995) boys. In Osijek-Baranja County, there were 64.2% (808) girls and 43.3% (559) boys who received at least one dose of the vaccine. The following results indicated that Osijek-Baranja County was one of the counties with the best proportion of children who received at least one dose of the vaccine [30].

Though there is strong evidence of HPV vaccination effectiveness, HPV vaccine hesitancy and low HPV-VCR remain significant challenges worldwide [10].

It is considered that several factors contribute to the relatively low HPV vaccination rate. These factors include negative environmental influences (i.e., the anti-vaxxer movement that spreads misinformation about vaccines or religious objections to vaccination, which can significantly influence the public perception and acceptance of the HPV vaccine), fear and concerns about potential side effects of the HPV vaccine, a lack of comprehensive education about HPV infection and vaccination, its potential health risks, or the benefits of vaccination, which can lead to a lower uptake of the vaccine [10,24,27].

Numerous studies have examined knowledge about HPV infection and vaccination worldwide. However, none of them has specifically focused on school populations and the impact of health education on their knowledge. In most of the research, the target population was young adults and students. The aforementioned research was conducted in countries such as India, Switzerland, Poland, and British Columbia [31,32,33,34].

A systematic literature review by Krokidi et al. found that health education interventions effectively increased the uptake, awareness, and acceptance of the HPV vaccine, aligning with our findings [31].

Recently published research by Brohman et al. tried to identify opportunities for HPV vaccine education by exploring the perspectives of students, parents, school staff, and public health nurses. In their research, parents were identified as the primary vaccine decision makers, which suggests that it is important to educate parents as well as students about vaccination against HPV [34].

Newly published research by Guarducci et al. focuses on the development of a network model to implement HPV vaccination coverage. The results obtained from their research, regardless of the limitations of the research, showed an increase in HPV vaccination coverage in both sex and in all observed cohorts. As the authors suggested, it is crucial to raise awareness about the importance of vaccination for all genders and to persist in advocating for a gender-neutral approach to HPV vaccination [35].

Research conducted by Miškulin et al. in 2021 among Croatian university students showed that the majority of students had satisfactory knowledge about HPV and the vaccination, but the vaccination uptake was very modest (only 20.8%) [36]. This research is one of the very few studies in Croatia regarding HPV knowledge and vaccination against HPV.

Our research, conducted among the school population in selected primary schools, showed better vaccination uptake results compared to the aforementioned studies. The overall vaccination rate was 62% for the entire school generation (which is an increase of 15% according to the previous generation), with 64% in the Control group and 65% in the Subject group. These findings suggest that our health education efforts positively influenced HPV vaccination coverage.

Regarding the self-assessment of one’s knowledge, the participants in the Subject group showed a significant progression following educational intervention. Prior to the education, participants in the Subject group rated their knowledge significantly lower than at any subsequent time point—immediately after the education, as well as 4 and 8 weeks later (Friedman test, *p* < 0.001). Moreover, after 8 weeks, the self-assessment of one’s knowledge in the group of students who completed the educational program (Subjects) demonstrated a significantly higher self-assessment of their knowledge compared to those in the Control group (Mann–Whitney U test, *p* < 0.001).

Finally, our subject group demonstrated a significant increase in knowledge about HPV infection and HPV vaccination following health education. This increase was observed not only immediately after the education session but also persisted at 4 weeks and 8 weeks post-education. These results indicate that health education has a positive impact not only on the vaccination coverage, but also on the self-assessment of one’s knowledge and knowledge related to HPV infection and vaccination.

Health education about the benefits of HPV vaccination is crucial and requires a comprehensive approach. Although it is important to educate parents, since they are the ones who decide to vaccinate their minor children, it is equally important (if not more important) to educate students on this topic, as they are increasingly more and more involved in making decisions related to their health.

There is still a paucity of the literature about the influence of health education on vaccination coverage and knowledge of the school population related to infection and vaccination against HPV in Croatia or the research about vaccination coverage.

This study has several limitations, such as using a convenient sample. Non-randomization could introduce selection bias. Future studies would benefit from blinding, randomization, a greater sample, and an analysis of a greater number of confounders. Longer follow-up would provide better insight into the durability of the educational impact on both knowledge retention and vaccination rates.

Therefore, further research is needed to establish and confirm a connection between the positive influence of health education on HPV vaccination coverage and knowledge related to vaccination and infection caused by the human papillomavirus in the studied population in Croatia.

## 5. Conclusions

HPV vaccination is the most effective way to prevent infection and the development of HPV-related diseases. However, there is still resistance to this vaccine among some parents and children, which is why vaccination should be popularized, the level of knowledge related to infection and HPV vaccination increased, and the awareness of the benefits of vaccination raised, concerning all diseases that HPV infection can potentially cause. Given that elementary school students are the vaccine target population, this goal could be achieved by improving the quality of their health education. Our research and the obtained results suggest the importance of health education since the students who completed the health education (Subjects) had better knowledge about vaccination and infection caused by HPV at all time points of assessment compared to the students participating in the Control group. Although there was an equal share of participants in both groups who were vaccinated against HPV, there was a visible increase in the share of vaccinated students within the entire generation (47% in the school year 2021/2022 vs. 62% in the school year 2022/2023) in the observed elementary schools, which could be the result of the implementation of this research.

Although a positive trend in a slight increase in the percentage of HPV vaccination has been observed in Osijek-Baranja County in recent years, additional efforts are still needed to achieve the highest possible rate of HPV vaccination in the target population. This goal could be achieved with the help of better and more effective health education of the target population as well as the popularization of the vaccination itself.

## Figures and Tables

**Table 1:** Age of subjects concerning groups.

	Arithmetic Mean (SD Standard Deviation)		Difference ^‡^	95% CI (Confidence Interval)	*p* *
	Control	Subjects			
Subject age (years)	13.7 (0.50)	13.7 (0.49)	−0.03	−0.18 to 0.12	0.69

* Mann–Whitney U test; ^‡^ Hodges–Lehmann median difference.

**Table 2:** Sex of subjects concerning groups.

	Number (%) of Subjects			*p* *
	Control	Subjects	Total	
Sex				
Male	52 (57%)	44 (56%)	96 (56%)	0.85
Female	39 (43%)	35 (44%)	74 (44%)	

* χ^2^ test.

**Table 3:** Distribution of student participants according to HPV vaccination by groups.

	Number (%) of Subjects			*p* *
	Control	Subjects	Total	
HPV vaccination—all				
Not vaccinated	33 (36.3%)	28 (35.4%)	61 (35.9%)	0.91
Vaccinated	58 (63.7%)	51 (64.6%)	109 (64.1%)	

* χ^2^ test.

**Table 4:** Distribution of student participants according to vaccination by sex.

	Number (%) of Subjects			*p* *
	Not Vaccinated	Vaccinated	Total	
Sex				
Male	42 (69.0%)	54 (49.5%)	96 (57.0%)	0.02
Female	19 (31.0%)	55 (50.5%)	74 (43.0%)	

* χ^2^ test.

**Table 5:** Vaccination of all students during the school year 2021/2022 and 2022/2023.

	Number (%) of Students in the Generation			
	2021/2022		2022/2023	
	Total	Vaccinated	Total	Vaccinated
School				
Ivan Filipović Elementary School	48	17 (35%)	61	42 (69%)
Retfala Elementary School	61	30 (49%)	62	36 (58%)
Višnjevac Elementary School	58	26 (45%)	59	31 (53%)
Josipovac Elementary School	29	19 (66%)	39	28 (72%)
Total	196	92 (47%)	221	137 (62%)

**Table 6:** Vaccination rate of students who participated in this research.

	Number (%)	
	Total	Vaccinated
Students—no education completed (Controls)	91	58 (64%)
Students—with education completed (Subjects)	79	51 (65%)

**Table 7:** Difference in self-assessment of one’s knowledge concerning groups.

	Median (Interquartile Range)		Difference ^‡^	95% CI (Confidence Interval)	*p* *
	Controls	Subjects			
Self-assessment of one’s knowledge	2 (1–2)	1 (1–2)	0	−1 to 0	0.04

* Mann–Whitney U test; ^‡^ Hodges–Lehmann median difference.

**Table 8:** Self-assessment of one’s knowledge during the entire observed period.

	Median (Interquartile Range) in the Subject Group				*p* *
	Before Education (0)	After Education (1)	4 Weeks After Education (2)	8 Weeks After Education (3)	
Self-assessment of one’s own knowledge	6 (4–8)	11 (9–12)	9 (8–12)	10 (8–12)	<0.001 ^†^

* Friedman test (Conover post hoc). ^†^ at level *p* < 0.013 (adjusted *p* value) significant difference (0) vs. (1, 2, 3); (1) vs. (2, 3).

**Table 9:** Self-assessment of knowledge in the control group and the subject group after 8 weeks.

	Number (%) of Student Participants					
	Insufficient	Poor	Good	Very Good	Excellent	Total
Controls						
According to your estimation, how much do you think you know about HPV and cervical cancer?	25 (27%)	39 (43%)	22 (24%)	5 (5%)	0	91 (100%)
According to your estimation, how much do you think you know about the severity of cervical cancer?	21 (23%)	31 (34%)	24 (26%)	10 (11%)	5 (5%)	91 (100%)
According to your estimation, how much do you think you know about the HPV vaccine?	19 (21%)	35 (38%)	21 (23%)	16 (18%)	0	91 (100%)
Subjects						
According to your estimation, how much do you think you know about HPV and cervical cancer?	9 (11%)	12 (15%)	31 (39%)	17 (22%)	10 (13%)	79 (100%)
According to your estimation, how much do you think you know about the severity of cervical cancer?	7 (9%)	16 (20%)	22 (28%)	18 (23%)	16 (20%)	79 (100%)
According to your estimation, how much do you think you know about the HPV vaccine?	7 (9%)	9 (11%)	34 (43%)	14 (18%)	15 (19%)	79 (100%)

**Table 10:** Self-assessment of one’s knowledge concerning groups eight weeks after education.

	Median (Interquartile Range)		Difference ^‡^	95% CI (Confidence Interval)	*p* *
	Controls	Subjects			
Assessment of one’s own knowledge	6 (5–9)	10 (8–12)	3	2 to 4	<0.001

* Mann–Whitney U test; ^‡^ Hodges–Lehmann median difference.

**Table 11:** The difference in the assessment of knowledge between the groups at “moment zero”.

	Median (Interquartile Range)		Difference ^‡^	95% CI (Confidence Interval)	*p* *
	Controls	Subjects			
Knowledge assessment (Caskey score)	9 (8–10)	9 (7–10)	0	−1 to 0	0.23

* Mann–Whitney U test; ^‡^ Hodges–Lehmann median difference.

**Table 12:** Knowledge assessment at three assessment points in the subject group.

	Number (%) by Correct Answer			*p* * (0 vs. 1)	*p* * (0 vs. 2)	*p* * (1 vs. 2)
	Before Education (0)	After Education (1)	4 Weeks After Education (2)			
HPV infection can cause herpes	40 (51%)	65 (82%)	41 (52%)	<0.001	>0.99	<0.001
If a woman or a girl has a normal PAP smear she doesn’t have HPV	22 (28%)	44 (56%)	42 (53%)	<0.001	0.001	0.85
HPV infection can cause genital warts	41 (52%)	74 (94%)	70 (89%)	<0.001	<0.001	0.34
A woman or a girl can get HPV infection from having sex	67 (85%)	76 (96%)	72 (91%)	0.02	0.27	0.34
HPV infection can cause cervical cancer	64 (81%)	73 (92%)	70 (89%)	0.02	0.24	0.55
HPV can be diagnosed during a gynecological or pelvic exam	57 (73%)	70 (89%)	59 (75%)	0.01	0.85	0.02
HPV can be treated with antibiotics	47 (59%)	62 (78%)	57 (72%)	0.006	0.08	0.46
HPV can be diagnosed by a blood test	21 (27%)	61 (78%)	47 (59%)	<0.001	<0.001	0.01
Girls/women who receive the HPV vaccine need less frequent pelvic exams	51 (65%)	56 (71%)	61 (77%)	0.47	0.10	0.41
Girls/Women who receive the HPV vaccine do not have to get PAP smears	58 (73%)	65 (82%)	64 (81%)	0.21	0.29	>0.99
The HPV vaccine protects against all sexually transmitted infections	47 (59%)	54 (68%)	58 (73%)	0.21	0.05	0.54
The HPV vaccine protects against cervical cancer	56 (71%)	72 (91%)	64 (81%)	<0.001	0.15	0.08
Girls/women who receive the HPV vaccine can worry less about getting any sexually transmitted infections	25 (32%)	35 (44%)	45 (57%)	0.05	0.001	0.08
Women who receive the HPV vaccine no longer have to use condoms during sexual intercourse.	61 (77%)	68 (86%)	69 (87%)	0.12	0.10	>0.99

* McNemar—Bowker test.

**Table 13:** Assessment of knowledge concerning all assessment points.

	Median (Interquartile Range) in the Subject Group				*p* *
	Before Education (0)	After Education (1)	4 Weeks After Education (2)	8 Weeks After Education (3)	
Knowledge assessment (Caskey score)	9 (7–10)	11 (10–13)	10 (9–12)	11 (9–12)	<0.001 ^†^

* Friedman test (Conover post hoc). ^†^ at level *p* < 0.013 (adjusted *p* value) significant difference (0) vs. (1, 2, 3); (0) vs. (2); (0) vs. (3).

**Table 14:** Difference in knowledge assessment according to groups after 8 weeks.

	Median (Interquartile Range)		Difference ^‡^	95% CI (Confidence Interval)	p *
	Controls	Subjects			
Knowledge assessment (Caskey score)	9 (8–10)	11 (9–12)	1	1 to 2	<0.001

* Mann–Whitney U test; ^‡^ Hodges–Lehmann median difference.

## Data Availability

All data are available and can be delivered to anyone upon request.
